# Distinct promoter methylation profile reveals spatial epigenetic heterogeneity in 2 myeloma patients with multifocal extramedullary relapses

**DOI:** 10.1186/s13148-018-0597-6

**Published:** 2018-12-20

**Authors:** Qiumei Yao, Gareth J. Morgan, Chor Sang Chim

**Affiliations:** 1 Department of Medicine, Queen Mary Hospital, The University of Hong Kong, Pokfulam Road, Pokfulam, Hong Kong; 2 Myeloma Institute, University of Arkansas for Medical Sciences, Little Rock, AR 72205 USA

**Keywords:** Multiple myeloma, Epigenetics, Tumor spatial heterogeneity

## Abstract

Spatial and subclonal genetic heterogeneity in multiple myeloma (MM) have been demonstrated by sequencing of plasma cells from multi-focal regions, but studies of spatial epigenetic heterogeneity are scanty. Herein, promoter methylation status of genes implicated in disease progression (CDKN2A and SHP1) and marrow escape (CDH1, CD56, and CXCR4) was studied in two patients with multi-focal extramedullary relapses. Patient 1 developed simultaneous chest wall and duodenal plasmacytoma at relapse. While SHP1 and CDKN2A were hypermethylated in both plasmacytomas, CDH1 hypermethylation was detected only in the chest wall. In patient 2, SHP1 methylation was found in the extradural plasmacytoma but not bone marrow (BM) at diagnosis, and the circulating PCs but not the BM at relapse. As the clonality, based on sequence of the complementarity-determining region 3 (CDR3) of the immunoglobulin gene, was conserved in plasma cells at diagnosis and relapse, differential methylation of CDH1 in patient 1 and SHP1 in patient 2 was an illustration of spatial epigenetic heterogeneity. Furthermore, subclonal epigenetic heterogeneity was identified by the presence of subclonal SHP1 promoter methylation within the chest wall plasmacytoma of patient 1. In summary, our data showed distinct promoter methylation profile of plasma cells from multiple regions. This is the first report of spatial epigenetic heterogeneity in MM.

**Electronic supplementary material:**

The online version of this article (10.1186/s13148-018-0597-6) contains supplementary material, which is available to authorized users.

## Main text

Intra-clonal heterogeneity in multiple myeloma (MM) is a requisite for clonal evolution and subsequent disease progression [1]. Deep sequencing of plasma cells (PCs) at different stages of disease and multiple sites revealed widespread intra-clonal and spatial genetic heterogeneity in MM [2, 3]. Promoter DNA methylation is an alternative mechanism of gene inactivation has been implicated in myeloma disease progression with prognostic impact [4–6]. However, study of spatial epigenetic heterogeneity is scanty. Herein, after showing identical clonality of PCs at diagnosis, and intra- and extra-medullary relapses, promoter methylation profiles of multiple genes were studied in two patients with multi-focal extramedullary relapses. These genes included tumor suppressor (SHP1 and CDKN2A), adhesion molecules (CDH1 and CD56), and chemokine receptor CXCR4. This is the first report of spatial epigenetic heterogeneity in MM.

**Fig. 1:**
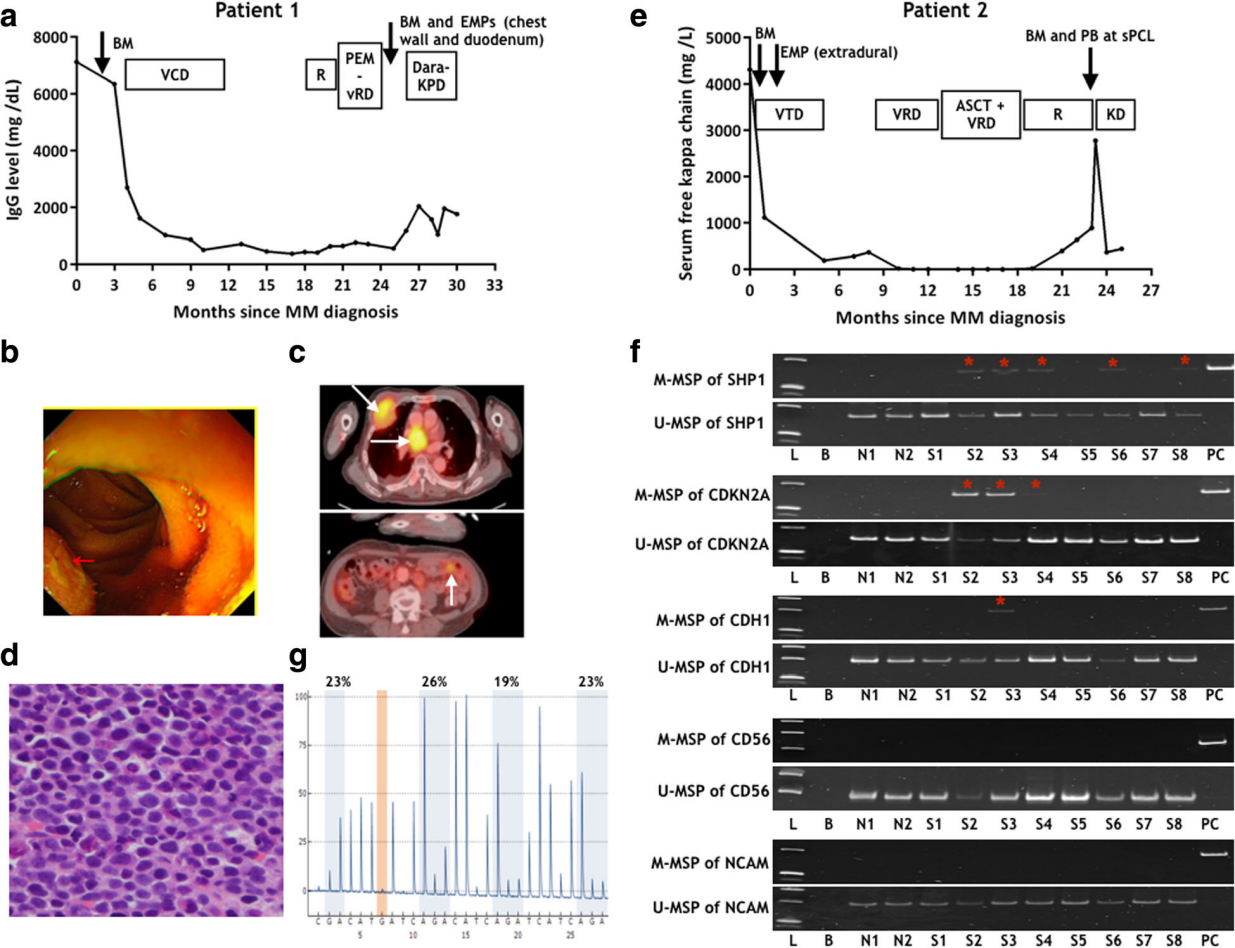
Disease progression and aberrant gene methylation in the two patients with multiple extramedullary diseases. **a** Disease course of patient 1 depicted by the response of immunoglobin (Ig) G and timing of tissue/bone marrow (BM) sampling. **b** Upper endoscopy of patient 1 showed polypoid lesion in duodenum (arrowed). **c** PET-CT of patient 1 showed multiple hypermetabolic extramedullary plasmacytomas (EMP) in the right anterior third rib, mediastinal and mid-jejunum causing intussusception (arrowed). **d** Histology of biopsy of chest wall mass of patient 1 showing exclusively infiltration with pleomorphic hyperchromatic plasma cells (H&E stain, original magnification 400×). **e** Disease course of patient 2 depicted by the response of free kappa light chain. **f** Methylation-specific polymerase chain reaction (MSP) study of SHP1, CDKN2A, CDH1, CD56, and CXCR4. L: DNA Ladder; B: bank; N1 and N2: normal marrow DNA; S1–4: diagnostic BM, duodenal plasmacytoma, chest wall plasmacytoma, paired BM of patient 1; S5–8: diagnostic BM, extradural plasmacytoma, paired BM and peripheral blood at secondary plasma cell leukemia (sPCL) of patient 2; PC: positive control of methylated DNA. In patient 1, SHP1 and CDKN2A were methylated at two EMPs and the paired bone marrow. Methylation of E-CAD was detected only in the chest wall plasmacytoma. In patient 2, methylation of SHP1 occurred in extradural plasmacytoma (2 months after diagnosis) and peripheral blood at the time of sPCL. DNA integrity was demonstrated by positive amplification of unmethylated-MSP of SHP1. **g** Pyrosequencing analysis of SHP1 in the chest wall plasmacytoma of patient 1 showing a mean CpG methylation of 23%. Abbreviations: VCD: bortezomib, cyclophosphamide and dexamethasone; pem-VRD: pembrolizumab-bortezomib-lenalidomide-dexamethason; Dara-KPD: daratumumab, carfilzomib, pomalidomide, dexamethasone; VTD: bortezomib, thalidomide, dexamethasone; ASCT: autologous stem cell transplantation. _*_: methylated MSP products were all verified by Sanger sequencing. Sequences are shown in Additional file 1: Figure S2

Patient 1 was a 68-year-old man with international stage (ISS) 2 IgGK MM without high-risk cytogenetic aberrations [t(4;14); t(14;16); del(17p)]. He achieved very good partial response (VGPR) with VCD (bortezomib-cyclophosphamide-dexamethasone), followed by lenalidomide maintenance. Two months into maintenance, he presented with left-sided sciatica due to a protruding L5 osseous plasmacytoma. Bone marrow (BM) then did not show plasmacytosis with a stable paraprotein of 2 g/L (Fig. 1a). After resolution of sciatica with irradiation, vRD (bortezomib-lenalidomide-dexamethasone) was started since Nov 2016 but he developed tarry stool with a chest swelling in Feb 2017. Upper endoscopy showed multiple polypoid lesions in the stomach and duodenum (Fig. 1b). Positron emission tomography–computed tomography (PET-CT) showed hypermetabolic extramedullary plasmacytoma (EMP) in the right anterior third rib, mediastinum, and mid-jejunum (Fig. 1c). Biopsy of chest wall mass showed exclusive malignant plasma cell infiltration (Fig. 1d). Biopsy of duodenal polyps showed plasmacytoma with diffuse infiltration of the lamina propria by CD138+ve pleomorphic PCs. However, there was only minimal rise of IgG paraprotein from 2 to 3 g/L, and BM yielded only 1% of PCs. He died of refractory myeloma despite Dara-KPD (daratumumab-carfilzomib-pomalidomide-dexamethasone).

Patient 2 was a 59-year-old woman with ISS-3 kappa light-chain MM. PET-CT at diagnosis did not reveal any extramedullary disease. Fluorescence in situ hybridization (FISH) showed amp(1q) but not high-risk cytogenetics. Two months into induction with VTD (bortezomib-thalidomide-dexamethasone), she developed cord compression, due to an extradural tumor at the C4 level, necessitating surgical decompression. Histology showed infiltration of the dura by sheets of CD138+ve pleomorphic PCs. She achieved CR but progressed again with multiple EMPs in the mediastinum, pleura, and paraspinal region 8 months later. She received VRD (bortezomib-lenalidomide-dexamethasone) re-induction, followed by autologous stem cell transplantation (ASCT) at VGPR (8/2016), VRD consolidation, and lenalidomide maintenance. Twelve months after ASCT, she developed aggressive disease progression with rapid doubling of serum-free kappa level (Fig. 1e), extensive BM infiltration (BMPC 90%), and secondary PC leukemia (sPCL) with 34% circulating PCs. Paired bone marrow and peripheral blood were collected at the time of sPCL.

DNA was extracted from diagnostic and relapsed BMs, EMP tissues, and circulating PCs at sPCL. Clonality, based on the sequences of complementarity-determining region 3 (CDR3), were identified by sequential PCR of immunoglobin (Ig)H VDJ, IgH DJ, and IgK VJ rearrangements [7]. Methylation-specific polymerase chain reaction (MSP) was performed as previously described with M- and U-MSP primers (Additional file 1: Table S1) [8]. Methylated-MSP (M-MSP) products were verified by Sanger sequencing. Quantitative pyrosequencing was performed for SHP1 (primers in Additional file 1: Table S1).

## Results and discussion

Clonality of the BMPC, based on the CDR3 sequence of the rearranged immunoglobulin (Ig) gene, were identical in all the samples including diagnostic BM, relapsed BM & EMP of each patient (Additional file 1: Table S2), consistent with previous finding that the CDR3 of the myeloma clone at diagnosis remains unchanged during disease progression [9].

While spatial genetic heterogeneity between intra- and extra-medullary myeloma has been demonstrated, there is as yet no data on spatial epigenetic heterogeneity. Therefore, we studied the methylation profile of five genes implicated in disease progression (SHP1, CDKN2A) or BM escape (CDH1, CD56, and CXCR4) by a highly sensitive MSP (10^− 3^ for both SHP1 and CDH1) (Additional file 1: Figure S1). At diagnosis, none of the five genes (SHP1, CDKN2A, CDH1, CD56, and CXCR4) were methylated in the diagnostic BMPC of patients 1 and 2 (Fig. 1f). However, promoter methylation of SHP1, CDKN2A, and CDH1 was observed in multiple relapsed/progressed samples (Fig. 1f), all verified by Sanger sequencing (Additional file 1: Figure S2). The change of pattern of methylation of these five genes is summarized in Table 1. Indeed, acquisition of methylation of these genes in BMPC or EMP at relapse is consistent with our previous observation that disease progression was marked by aberrant gene methylation [4].

**Table 1:** Patients with multiple extramedullary diseases

Patient	Time (months)	Sample	Plasma cell (%)	SHP1	CDKN2A	CDH1	CD56	CXCR4
1	0	Diagnostic BM	10	–	–	–	–	–
	26	Duodenal plasmacytoma	> 90	+	+	–	–	–
	26	Chest wall plasmacytoma	100	+	+	+	–	–
	26	Relapsed BM	1	+	+	–	–	–
2	0	Diagnostic BM	CD138 sorted cells	–	–	–	–	–
	2	Extradural plasmacytoma	> 90	+	–	–	–	–
	23	Relapsed BM	CD138 sorted cells	–	–	–	–	–
	23	Peripheral blood	34	+	–	–	–	–

*Abbreviations:* *BM* bone marrow; +, presence of methylation; −, absence of methylation

Patient 1 developed simultaneous chest wall and duodenal plasmacytoma at relapse. The five genes were completely unmethylated in the BM at diagnosis. While SHP1 and CDKN2A were hypermethylated in all sites (relapsed BM, and chest wall and duodenal plasmacytoma), CDH1 hypermethylation was only detected in the chest wall (Table 1). Given that all PCs were derived from the same clone with identical CDR3, the distinct methylation profile of the chest wall plasmacytoma from other anatomical sites revealed spatial epigenetic heterogeneity in MM. In patient 2, the extradural plasmacytoma occurred only 2 months after diagnosis. Despite the absence of EMP by PET-CT at diagnosis, this clone of PCs was likely present at the time of diagnosis. Therefore, the presence of SHP1 methylation in the extradural plasmacytoma but not BM at diagnosis was another example of spatial epigenetic heterogeneity. This spatial epigenetic heterogeneity was further illustrated at relapse by the presence of SHP1 methylation in the circulating PCs but not the BM at the time of sPCL. Moreover, in whole genome sequencing of PCs collected from iliac crest and radiology-guided focal lesions, spatial genetic heterogeneity was observed in more than 75% MM patients [3]. Spatial epigenetic heterogeneity has been demonstrated in metastatic squamous cell carcinoma of the esophagus by multi-region genome-wide methylation profiling, in which multiple tumor suppressor genes were found differentially methylated in different sites of metastasis [10]. By contrast, there is as no data about spatial epigenetic heterogeneity in MM.

Furthermore, while pathology examination of the chest wall plasmacytoma of patient 1 showed exclusively PCs (Fig. 1d), the percentage of SHP1 promoter methylation was 23% only by quantitative pyrosequencing (Fig. 1g). The presence of promoter DNA methylation within a subset of tumor cells is consistent with subclonal DNA methylation, hence a form of subclonal epigenetic heterogeneity, similar to clonal genetic heterogeneity in MM as evidenced by presence of gene mutations in subclonal populations [2].

Besides, in patient 2, SHP1 promoter methylation was detected in the extradural plasmacytoma of the spinal cord but not the diagnostic BM, and in the circulating PCs but not the paired BM at relapse. We had reported previously that aberrant methylation of SHP1 was associated with absent SHP1 expression and led to constitutive activation of the Jak/ STAT pathway [8]. However, the actual mechanism of methylation of SHP1 in EM myeloma remains to be defined.

In summary, our data showed that extramedullary relapses arise from the same clonal PCs at diagnosis. Spatial and subclonal epigenetic heterogeneity is present in MM. Since MSP only detects few CpG sites, genome-wide epigenetic study of intra- and extra-medullary myeloma at diagnosis and relapse is warranted.

## Additional file

Additional file 1: Materials and methods. **Table S1.** Primer sequences. **Table S2.** Clonality detected in the two patients with multiple extramedullary diseases. **Figure S1,** Assay of sensitivity of SHP1 and CDH1 by methylated methylation-specific polymerase chain reaction of the methylated positive control. **Figure S2.** Sequencing of methylated methylation-specific polymerase chain reaction (M-MSP) products. **Figure S3.** Methylation-specific polymerase chain reaction (MSP) study of SHP1 in normal peripheral blood. (DOCX 3973 kb)

## Abbreviations

BM: Bone marrow
CDR3: Complementarity-determining region 3
EMP: Extramedullary plasmacytoma
Ig: Immunoglobulin
ISS: International stage system
MM: Multiple myeloma
PCs: Plasma cells
PET-CT: Positron emission tomography–computed tomography
sPCL: Secondary PC leukemia
VCD: Bortezomib-cyclophosphamide-dexamethasone
VGPR: Very good partial response
VTD: Bortezomib-thalidomide-dexamethasone
