# Supplementary material for: Distinct promoter methylation profile reveals spatial epigenetic heterogeneity in 2 myeloma patients with multifocal extramedullary relapses
Source: Clin Epigenetics. 2018 Dec 20;10:158. doi: 10.1186/s13148-018-0597-6 (PMC6302381; doi:10.1186/s13148-018-0597-6)
Supplement: Supplementary file 1 — Materials and methods. Table S1. Primer sequences. Table S2. Clonality detected in the two patients with multiple extramedullary diseases. Figure S1, Assay of sensitivity of SHP1 and CDH1 by methylated methylation-specific polymerase chain reaction of the methylated positive control. Figure S2. Sequencing of methylated methylation-specific polymerase chain reaction (M-MSP) products. Figure S3. Methylation-specific polymerase chain reaction (MSP) study of SHP1 in normal peripheral blood. (DOCX 3973 kb) [file 13148_2018_597_MOESM1_ESM.docx]

**Distinct promoter methylation profile reveals spatial epigenetic heterogeneity in 2 myeloma patients with multifocal extramedullary relapses**

**Materials and methods**

Sensitivity of MSP

One microgram of DNA of the methylation positive control (Merck KGaA, Darmstadt, Germany) was 10-fold diluted serially in normal marrow DNA, modified by bisulfite and then amplified by M-MSP as previously published [1].

**Table S1 Primer sequences**

|  | **Forward (5’ to 3’)** | **Reverse (5’ to 3’)** | **Reference** |
| --- | --- | --- | --- |
| Methylation-specific PCR | |  |  |
| SHP1 M-MSP | GAA CGT TAT TAT AGT ATA GCG TTC | TCA CGC ATA CGA ACC CAA ACG | [2] |
| CDKN2A M-MSP | TTA TTA GAG GGT GGG GCG GAT C | GAC CCC GAA CCG CGA CCG TAA |  |
| CDH1 M-MSP | GTG GGC GGG TCG TTA GTT TC | CTC ACA AAT ACT TTA CAA TTC CGA CG |  |
| CD56 M-MSP | GGT CGC GTT TTG TAG GTT TTC | CTT ACG CTA ACC CGA ATT CG |  |
| CXCR4 M-MSP | TTA TTT ATT TTA GTA AGG ATG GAC GC | AAA AAC CCT ACT ATT TAC GAA TAA TCG |  |
| SHP1 U-MSP | GTG AAT GTT ATT ATA GTA TAG TGT TTG G | TTC ACA CAT ACA AAC CCA AAC AAT | [2] |
| CDKN2A U-MSP | TTA TTA GAG GGT GGG GTG GAT T | CAA CCC CAA ACC ACA ACC ATA A |  |
| CDH1 U-MSP | GGT GGG TGG GTT GTT AGT TTT GT | AAC TCA CAA ATA CTT TAC AAT TCC AAC A |  |
| CD56 U-MSP | TTT TAG GTT GTG TTT TGT AGG TTT TT | AAA TCC TTA CAC TAA CCC AAA TTC A |  |
| CXCR4 U-MSP | GAA TTT TAT TTA TTT TAG TAA GGA TGG ATG T | CAA AAA ACC CTA CTA TTT ACA AAT AAT CA |  |
| Methylation analysis of SHP1 by Pyrosequencing | |  |  |
| PCR | AGGGTTGTGGTGAGAAATTAATTAG (with 5′ Biotin) | ACACTCCAAACCCAAATAATACTTC |  |
| Sequencing primer | | AACCCAAATAATACTTCAC |  |

**Table S2 Clonality detected in the 2 patients with multiple extramedullary diseases**

| patient | Time (months) | sample |  | Clonality |
| --- | --- | --- | --- | --- |
| 1 | 0 | bone marrow | Clonotype: V_H_4.59(0)-8-(5)D3.22(10)-12-(3)J_H_3  CDR3 sequences:  gcgagagatgaggctaactatgattatagtggccccccccgatttgctttggatatc | |
|  | 26 | bone marrow |  |  |
|  | 26 | chest wall plasmacytoma |  |  |
|  | 26 | duodenal plasmacytoma |  |  |
|  |  |  |  |  |
| 2 | 0 | bone marrow | Clonotype: V_K_1.39-0-(0)-0-J_K_5  CDR3 sequences  caacagacttacagtacccctccgacaacc | |
|  | 2 | extradural plasmacytoma |  |  |
|  | 23 | bone marrow |  |  |
|  | 23 | peripheral blood |  |  |

**Figure S1 Assay of sensitivity of SHP1 and CDH1 by methylated methylation-specific polymerase chain reaction of the methylated positive control.** B: bank; N: normal marrow DNA; 1x: undiluted methylated positive control; 10^-1^ to 10^-4^: serial dilution of methylated control DNA from 10^-1^ to 10^-4^.


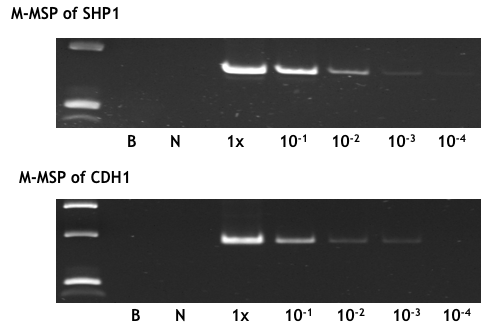


**Figure S2** **Sequencing of methylated methylation-specific polymerase chain reaction (M-MSP) products.** Methylated positive controls (PC) and all methylated primary samples (S) were verified by sequencing. The DNA sequence of the “methylated” MSP product was compared with the germ line sequence of the wild-type DNA (WT). Methylated cytosine residues in CpG dinucleotide remained as C, whereas unmethylated cytosine read as T after bisulfite conversion. From the top to the bottom on the left: M-MSP of SHP1 of PC, S2-4, S6 and S8. From the top to the bottom on the right: M-MSP of CDKN2A of PC, S2-4 and M-MSP of CDH1 of PC, S3. PC: positive control of methylated DNA; S2-4: duodenal plasmacytoma, chest wall plasmacytoma, relapsed bone marrow of patient 1; S6: extradural plasmacytoma of patient 2; S8: circulating plasma cells at the time of secondary plasma cell leukemia of patient 2.


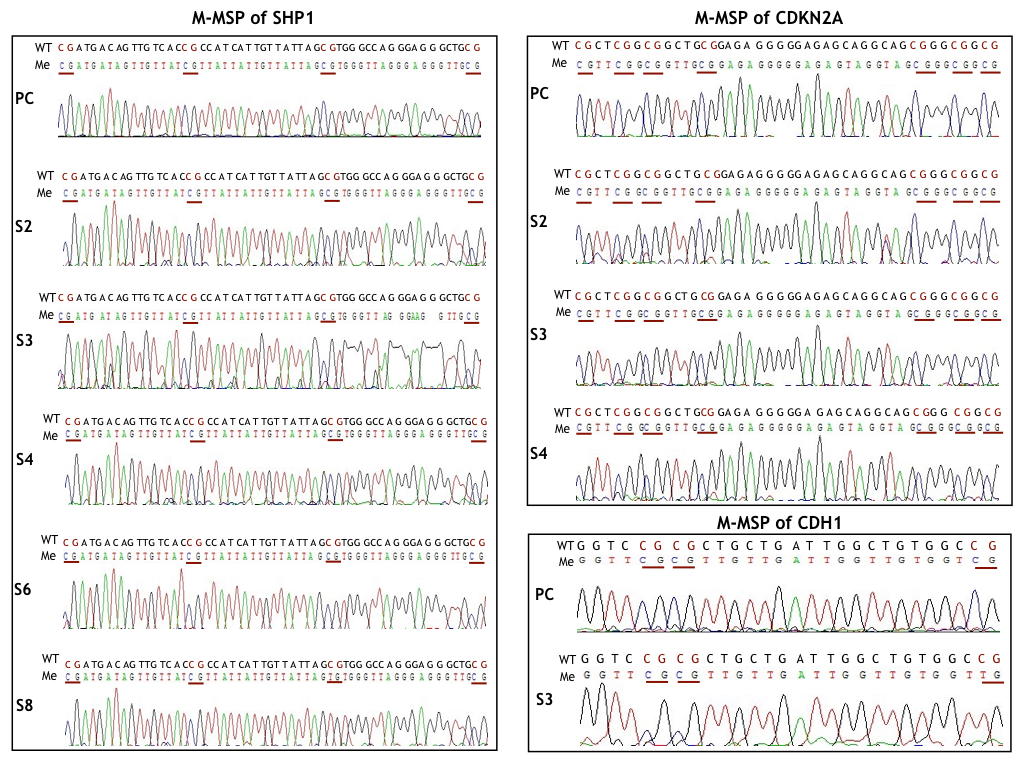


**Figure S3.** Methylation-specific polymerase chain reaction (MSP) study of SHP1 in normal peripheral blood. L: DNA Ladder; B: bank; N1-N2: normal peripheral blood DNA; PC: positive control of methylated DNA; M-MSP: methylated MSP; U-MSP: unmethylated MSP.


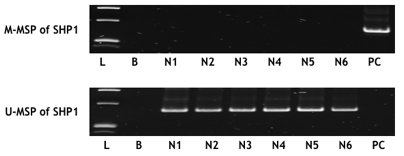


**References**

1. Chim CS, Kwong YL, Fung TK, Liang R. Methylation profiling in multiple myeloma. Leukemia research. 2004;28:379-85.

2. Chim CS, Liang R, Leung MH, Kwong YL. Aberrant gene methylation implicated in the progression of monoclonal gammopathy of undetermined significance to multiple myeloma. J Clin Pathol. 2007;60:104-6.
